# CREB5 regulates stem cell-like transcriptional programs to enhance tumor progression in prostate cancer

**DOI:** 10.18632/oncotarget.28850

**Published:** 2026-03-17

**Authors:** Allison Makovec, John T. Phoenix, Hannah E. Bergom, Ella Boytim, Ava P. Gustafson, Aiden Deacon, Sydney Tape, Atef Ali, Megan Ludwig, Samuel P. Pitzen, David Moline, Camden Richter, Hudson Longie, Mei-Chi Su, Sampreeti Jena, Pornlada Likasitwatanakul, Justin M. Drake, R. Stephanie Huang, William C. Hahn, Jonathan P. Rennhack, Scott M. Dehm, Steven Kregel, Emmanuel S. Antonarakis, Justin Hwang

**Affiliations:** ^1^Department of Medicine, University of Minnesota - Twin Cities, Minneapolis, MN 55455, USA; ^2^Masonic Cancer Center, University of Minnesota - Twin Cities, Minneapolis, MN 55455, USA; ^3^University of Kansas Medical Center, Kansas City, KS 66160, USA; ^4^Department of Cancer Biology, Loyola University Chicago, Chicago, IL 60153, USA; ^5^Cancer Biology Graduate Program, University of Colorado Anschutz, Aurora, CO 80045, USA; ^6^Medical College of Wisconsin, Green Bay, WI 54115, USA; ^7^Bioinformatic Interdepartmental Program, University of California, Los Angeles, CA 90095, USA; ^8^Department of Pharmacology, University of Minnesota - Twin Cities, Minneapolis, MN 55455, USA; ^9^Graduate Program in Molecular, Cellular, and Developmental Biology and Genetics, University of Minnesota, Minneapolis, MN 55455, USA; ^10^Rush University Medical College, Chicago, IL 60612, USA; ^11^Department of Experimental and Clinical Pharmacology, University of Minnesota - Twin Cities, Minneapolis, MN 55455, USA; ^12^Department of Medicine, Siriraj Hospital, Mahidol University, Bangkok 10700, Thailand; ^13^Broad Institute of MIT and Harvard, Cambridge, MA 02142, USA; ^14^Department of Medical Oncology, Dana-Farber Cancer Institute and Harvard Medical School, Boston, MA 02215, USA; ^15^Department of Laboratory Medicine and Pathology, University of Minnesota-Twin Cities, Minneapolis, MN 55455, USA; ^16^Department of Urology, University of Minnesota-Twin Cities, Minneapolis, MN 55455, USA

**Keywords:** prostate cancer, CREB5, basal-like, stem cell-like, AP-1 transcription factors

## Abstract

Prostate gland cells can be transcriptionally and morphologically characterized as basal and luminal. About 30–40% of advanced prostate cancers (PC) harbor basal-like transcription programs. In castration-resistant PC (CRPC), studies indicate that basal and stem cell-like (SCL) tumors are major resistance mechanisms to androgen receptor (AR)-targeted therapies. SCL tumors have reduced AR activity and increased stem-cell activity that promotes tumor formation, which contributes to poor clinical outcomes. We determined that CREB5 is a key regulator of basal and SCL transcriptional programs and tumor-forming phenotypes in PC. Through *in silico* modeling of PC transcriptomes and several pre-defined PC signaling programs, *CREB5* expression was best associated with basal-like gene signatures and SCL-associated genes in primary PC and CRPCs (*n* = 493 and 208). This included associations with *FOSL1* and other AP-1 transcription factors. We further found that CREB5 interacted with AP-1 proteins and bound to the regulatory elements of AP-1 genes, suggesting a mechanistic role in regulating the activity of AP-1 genes. In AR-positive cells, *CREB5* overexpression promoted cell colony growth with tumorigenic properties and increased tumor size *in vivo*. These findings implicate CREB5 as a driver of the transcriptional programs underlying AR-independent basal and SCL CRPC subtypes, and this activity is detectable in primary PC.

## INTRODUCTION

Androgen receptor (AR) signaling is a key driver of prostate cancer (PC) progression and is a primary therapeutic target in both locally advanced and metastatic disease. AR pathway inhibitors (ARPIs), such as abiraterone and enzalutamide, form the foundation of treatment for castration-resistant prostate cancer (CRPC) [1]. While these treatments often extend survival, a considerable subset of tumors demonstrates either primary resistance or rapid progression despite AR-targeted therapy [2]. Of these resistant tumors, many exhibit diminished *AR* expression or activity and rely on alternative pathways for growth and survival [3]. Understanding the biology of AR-independent disease is critical for identifying new therapeutic vulnerabilities in these advanced PCs.

Our recent studies revealed that certain ARPI-resistant CRPCs [4] exhibit transcriptional profiles resembling basal, club, and hillock cell types, which are AR-negative cells generally found in the benign prostate [5]. When these transcriptional profiles are found in the setting of late-stage PC, this mixed-epithelial identity suggests a role for lineage plasticity as a mechanism of therapy resistance and disease progression [4, 5]. Our prior work distinguished these tumors as a unique subset of ARPI-resistant CRPCs that either lacked typical AR activity or had histopathological neuroendocrine characteristics [6], which are the more studied molecular subtypes of CRPCs. We instead found that the basal, club, or hillock signatures were associated with stem cell-like (SCL) transcriptional programs. Epigenetic profiling of CRPC models and clinical specimens by Tang et al. defined 4 major subtypes of CRPC, in which the second most common subtype exhibited an SCL program [7]. Distinct from the pluripotent stem cell program, this SCL transcriptional program found in CRPCs constitutes many AP-1 transcription factors such as FOSL1, a metastatic factor in pancreatic cancers and a poor prognostic factor in renal cell carcinomas that exhibit sarcomatoid differentiation [8, 9]. In regulating these transcriptional activities, our prior work also implicated that transcriptional regulator, such as Krüppel-like factor 5 (KLF5), were mechanistic drivers of these transcriptional programs [4].

Cyclic AMP response element-binding protein 5 (CREB5) is a transcription factor previously implicated in the disease progression of multiple cancers, including breast, colorectal, and prostate cancers [10–13]. In PC cell lines, overexpression of *CREB5* has been shown to reprogram chromatin binding of AR and FOXA1 and promote resistance to ARPIs [14]. Beyond AR interactions, across several cancer types, *CREB5* expression has been associated with stemness and differentiation [15]. CREB5 also appears to be a regulator of metastasis in other hormone-driven cancers, such as breast and ovarian [10, 11, 16], as well as non-hormonedriven cancers, like colorectal and brain tumors [15, 17]. Furthermore, in gliomas, CREB5 has been found to regulate cancer stem cell progression [18]. While these studies independently point to CREB5 as a key factor of tumor progression, the underlying transcriptional regulatory role of CREB5 remains elusive. Here, we investigate CREB5 as a potential upstream regulator of basal and SCL transcriptional programs in various stages of PC.

In this study, through both computational and molecular characterization of PC cell lines, we determined that *CREB5* is associated with basal PC and drives SCL traits. CREB5 interacted with activator protein-1 (AP-1) transcription factors, such as FOSL1, and regulated the expression of other AP-1 members at transcription regulatory elements. Understanding *CREB5* as a driver of AR-independent PC may help elucidate novel therapeutic targets to perturb CREB5 signaling in late-stage and ARPI-resistant cancers.

## RESULTS

### CREB5 expression is associated with basal-like profiles and inversely correlates with AR activity

**Figure 1:**
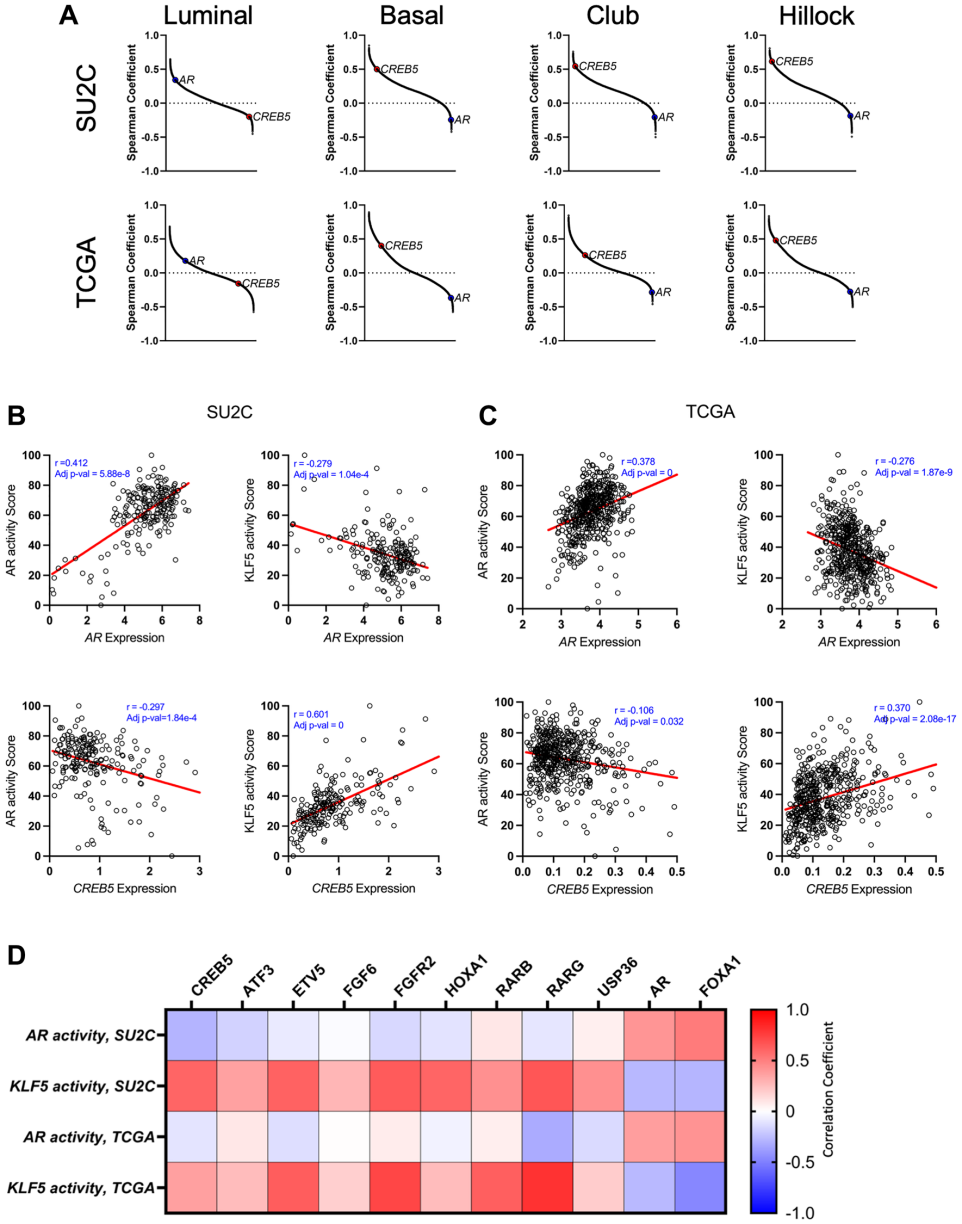
(**A**) Snake plots rank the gene correlations to luminal, basal, club and hillock signatures that were computed based on the transcriptomes of CRPC patient samples from SU2C (*n* = 208) and primary PC from TCGA (*n* = 493). Correlations between AR, CREB5, or KLF5 activity scores and *AR* or *CREB5* expression in each sample are depicted in scatterplots based on samples from (**B**) SU2C and (**C**) TCGA. (**D**) A heatmap shows the relative association between AR or KLF5 activity scores and a suite of genes that were shown to drive resistance in a previous ORF screen [11].

In prior genome-scale overexpression screens in prostate cancer cell lines, *CREB5* was a top-scoring gene implicated in resistance to androgen deprivation therapies (ADT) and ARPIs [14]. However, CREB5’s relationship with basal, club, and hillock gene expression signatures has not been explored in clinical specimens. As *AR* expression and activity define the luminal phenotypes in CRPC, we evaluated the association between *CREB5* and *AR* in CRPC samples from the Stand Up 2 Cancer (SU2C) dataset (*n* = 208) [19] and primary tumors from the Cancer Genome Atlas (TCGA, *n* = 493). By ranking all ~20,000 detectable genes based on their correlation with gene sets that define luminal, basal, club, and hillock epithelial cell identities, we found that *CREB5* ranked among the top genes associated with basal, club, and hillock identity but was among the lowest associated with luminal identity (Figure 1A, Table 1 and Supplementary Data 1). *AR* was expectedly positively correlated with luminal cells but negatively correlated with the other subtypes (Figure 1A). KLF5 is a transcription factor associated with ARPI resistance as well as basal, club, hillock, and SCL transcriptional subtypes [4]. Our next analyses indicated that KLF5 activity was positively correlated with *CREB5* in primary PC and CRPC (Figure 1B, 1C). Finally, KLF5 activity also correlated with enriched expression of other genes previously identified as ARPI resistance genes in PC cell lines through a genome-scale overexpression screen, including those encoding transcription factors (*ATF3*, *ETV5*), nuclear receptors (*RARB*, *RARG*), or regulators of FGF signaling (*FGF6*, *FGFR2*) [14]. KLF5 activity was expectedly anticorrelated with *AR* and *FOXA1*, an AR coregulator (Figure 1D and Supplementary Data 1). Overall, *CREB5* and other genes that promote ARPI resistance were transcriptionally associated with the basal cell subtype and anticorrelated with *AR* in CRPC. Intriguingly, these inverse relationships between the transcriptomes were found in both primary PC and CRPC.

**Table 1:** CREB5’s association with epithelial cell identity-related gene signatures

Signature	SU2C		TCGA	
	*CREB5* Spearman correlation coefficient (ρ)	*CREB5* Adj *p*-value	*CREB5* Spearman correlation coefficient (ρ)	*CREB5* Adj *p*-value
Basal	0.501	>0.001	0.400	>0.001
Club	0.544	>0.001	0.263	>0.001
Hillock	0.615	>0.001	0.479	>0.001
Luminal	−0.200	0.016	−0.155	0.001

Spearman correlation and adjusted *p*-values for the correlation between *CREB5* and luminal, basal, club, and hillock epithelial cell identities.

### Molecular associations of CREB5 in prostate tumors

**Figure 2:**
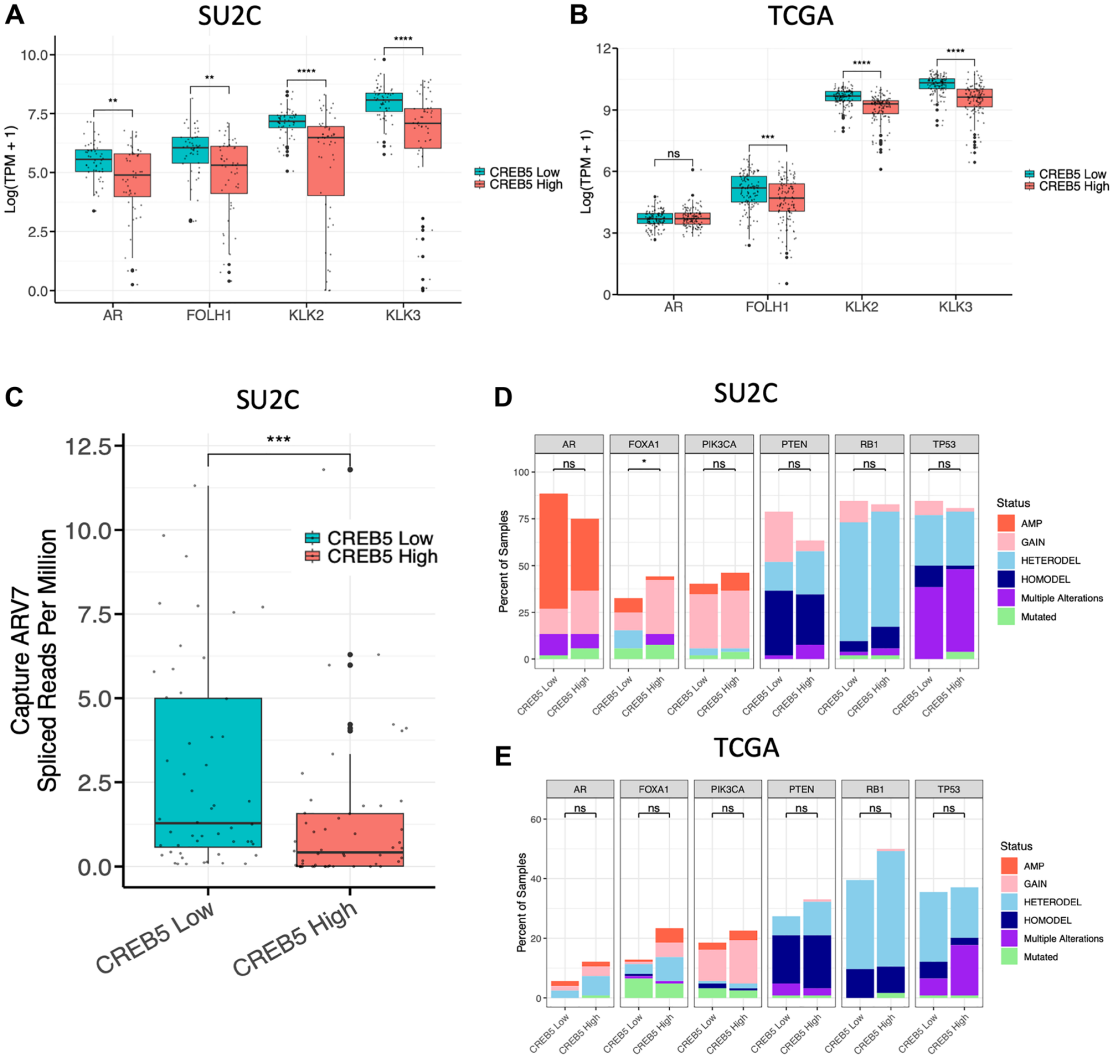
Relative gene expression of *AR*, *FOLH1* (PSMA), *KLK2*, and *KLK3* (PSA) are shown across the top (high) and bottom (low) quartiles of *CREB5* expression in (**A**) CRPC (SU2C, *n* = 104) and (**B**) primary PC tumors (TCGA, *n* = 493). (**C**) In the same CRPC cohort, we compared *AR-V7* expression levels in tumors with high and low *CREB5* expression. (**D**, **E**) Using stacked bar graphs, we further compared the percent of tumors with various types of somatic alterations in CRPC and primary PC tumors with high and low *CREB5* expression. Differences in alteration frequencies were evaluated using Fisher’s exact test (^*^p < 0.05, ^**^p < 0.01, ^**^p < 0.001, ^**^p < 0.0001, and ns = non-significant).

We next evaluated the molecular association of *CREB5* expression with respect to critical oncogenes and tumor suppressors [20, 21] with known pathogenic roles in prostate cancer. In CRPCs, high *CREB5* expression, as based on expression quartiles, conferred significantly lower expression levels of *AR*, *FOLH1* (PSMA), *KLK2*, and *KLK3* (PSA), as compared to *CREB5* low tumors (Figure 2A). With the exception of *AR* expression, primary PCs exhibited the same patterns (Figure 2B). *AR-V7* is a constitutively active *AR* splice variant that lacks the ligand-binding domain and therefore is not regulated by androgens. Therefore, increases in *AR-V7* drive therapy resistance in prostate cancer patients and cell models by bypassing the need for androgen binding, making it a key mechanism of resistance in CRPC [22, 23]. In CRPCs from the SU2C study, we found decreased AR-V7 expression in *CREB5*-high tumors compared with *CREB5-* low tumors (Figure 2C). We next examined several genomic alterations that have been frequently discussed in landscape studies of CRPCs [20, 21]. However, we found limited significant differences across the *CREB5* high and low tumors in both CRPC and primary PC (Figure 2D, 2E). Altogether, these findings based on transcriptomic analysis support that *CREB5* is inversely associated with the expression of *AR* and AR target genes but was not associated with key somatic drivers of CRPCs.

### CREB5 is associated with SCL genes, including FOSL1

**Figure 3:**
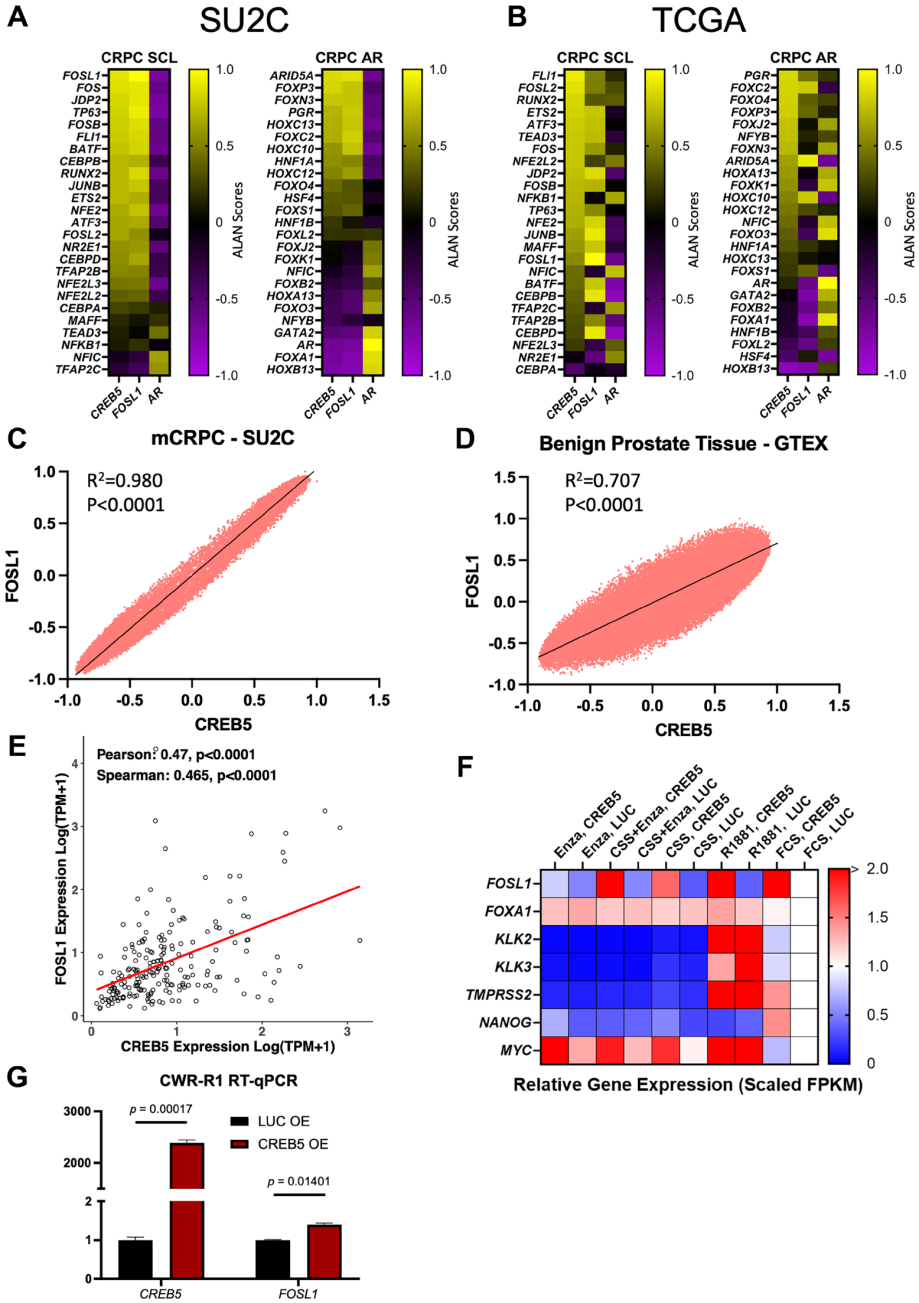
Gene behavior comparisons between CREB5, FOSL1, or AR and SCL, or AR CRPC signatures were established using the algorithm for linking activity networks (ALAN). Outputs ranging from −1 to 1 were organized into heatmaps for both (**A**) SU2C (*n* = 208) and (**B**) TCGA (*n* = 493). The ALAN interaction of *CREB5* and *FOSL1* was depicted for all genes in the (**C**) SU2C and (**D**) GTEx (*n* = 245) cohorts. (**E**) The gene expression of *CREB5* and *FOSL1* from SU2C samples is displayed in a scatterplot. (**F**) A heatmap depicts the ratio of expression of genes using RNA-seq data from LNCaP cells. Cells were grown in conditions containing one of the following or a combination: charcoal strip serum (CSS), Enzalutamide (Enza), fetal calf serum (FCS), and R1881. (**G**) Expression of *CREB5* and *FOSL1* in CWR-R1 cells, as determined by RT-qPCR, is displayed in bar graphs.

The role of CREB5 in transcriptional reprogramming has previously been linked to FOXA1 [13], which has separately been discussed to support the reactivation of development programs by CREB5 in prostate cancer [24]. To investigate the reprogramming processes in metastatic prostate cancer, we examined the relationship between *CREB5* and the CRPC subtypes defined via epigenetic profiling of CRPC models, as reported by Tang et al. [7]. To do this, we used the Algorithm for Linking Activity Networks (ALAN) [25] to compare gene behavior. Unlike gene-to-gene associations, ALAN considers the relationship of each gene with respect to all other genes across all samples. Thus, when examining 20,000 genes, ALAN can determine 400,000,000 potential interactions. Through ALAN, the gene behavior of *CREB5* was compared to two suites of 25 transcription factors that were used to characterize CRPCs into stem cell-like (SCL) or AR-active subtypes [7]. Here, we also compared *CREB5* to *FOSL1*, which was nominated as the top CRPC-SCL transcription factor based on Tang et al. Through this analysis of CRPCs (*n* = 208), *CREB5*’s behavior was largely associated with the 25 SCL transcription factors and exhibited very similar patterns to *FOSL1*. Furthermore, both *CREB5* and *FOSL1* were anticorrelated with the behavior of AR (Figure 3A, 3B). While the SCL and AR subtypes defined by their 25 transcription factors are optimized for CRPC, we also evaluated *CREB*5’s association with these genes in primary PC (TCGA, *n* = 493). In primary PC, we found similar results as in CRPCs. In this analysis, when examining the 25 transcription factors associated with the CRPC AR subtype, the alignments of *CREB5* and *FOSL1* exhibited less alignment with the 25 transcription factors in the CRPC SCL subtype (Figure 3A, 3B and Supplementary Data 1).

As *FOSL1* is a SCL gene that was recently implicated in the metastasis of pancreatic and is a poor prognostic factor in renal cancers with sarcomatoid differentiation [8, 9], we expanded upon this finding and determined via ALAN that *CREB5* exhibited highly concordant behavior with *FOSL1* across all potential interactions in both CRPC (*n* = 208, R^2^ = 0.980) and benign prostate tissue from the GTEx dataset (*n* = 245, R^2^ = 0.707), but had the strongest alignment in CRPC. (Figure 3C, 3D). *FOSL1* expression levels were further associated with *CREB5* expression across this CRPC cohort (r = 0.47, ρ = 0.47, *p* < 0.001; Figure 3E). In PC cell lines (LNCaP), through RNA-sequencing, we found that *CREB5* overexpression promoted higher *FOSL1* expression than overexpression of luciferase (*LUC*), an inert negative control gene (Figure 3F). This result was consistent across treatment conditions; cells subjected to charcoal-stripped serum (ADT mimic), enzalutamide (AR-antagonist), and R1881 (AR-agonist) all showed that *CREB5* overexpression increased *FOSL1* expression relative to the control *LUC* overexpression. In culture with both CSS and enzalutamide, *FOSL1* expression was decreased, but to a lesser extent in *CREB5* compared to *LUC* overexpressing cells (Figure 3F). When stimulating AR activity in cell cultures through the androgen analog R1881, we once again observed that *CREB5* overexpression increased *FOSL1* expression (Figure 3F). To further corroborate the relationship between *CREB5* and *FOSL1*, we overexpressed *CREB5* in an additional cell line, CWR-R1, which models CRPC. Here, we found that *CREB5* overexpression significantly increased *FOSL1* expression (*p* = 0.014) through RT-qPCR (Figure 3G and Supplementary Data 1). These collective observations suggest that *CREB5* expression and activity are associated with certain AP-1 transcription factors, such as *FOSL1*, which promote SCL traits.

### CREB5 regulates AP-1 transcription factors

**Figure 4:**
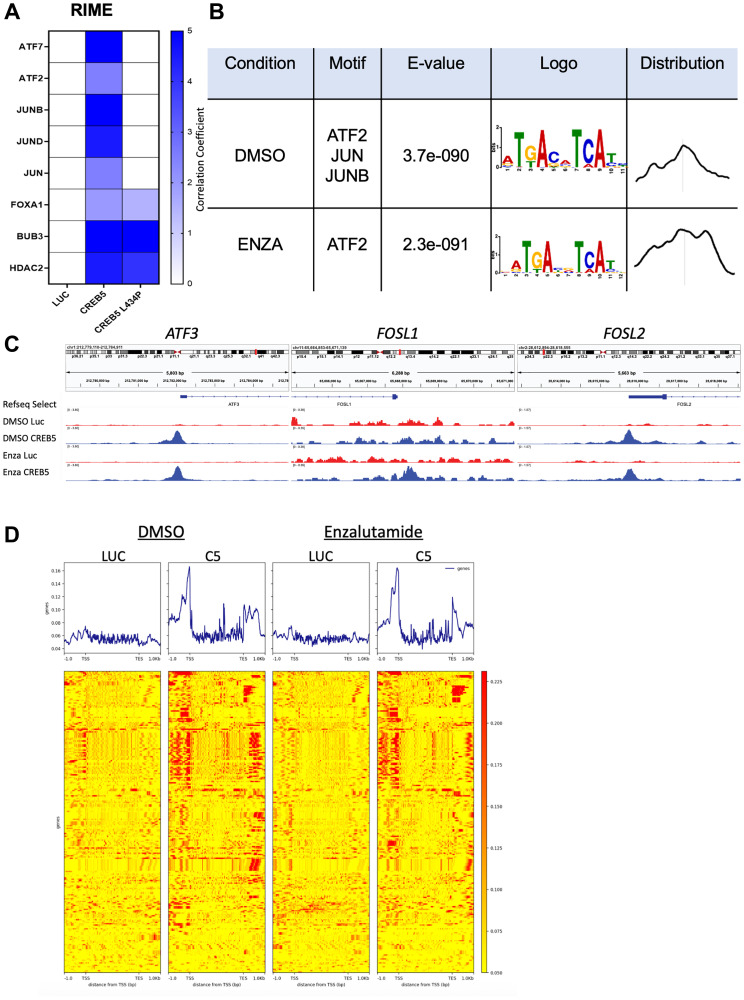
(**A**) RIME interactions in LNCaP cells with either CREB5 or CREB5 LA43P overexpressed are shown relative to luciferase (LUC) overexpressing cells via a heatmap, in which key AP-1 proteins are indicated. (**B**) Motif enrichment analysis was conducted based on CREB5 binding sites derived from ChIP-seq conducted in LNCaP cells overexpressing CREB5 cultured in either Enzalutamide (Enza) or DMSO, as shown in a table. (**C**) CREB5 binding status at transcriptional start sites of ATF3, FOSL1, and FOSL2 in CREB5 overexpressed LNCaP cells vs. LUC overexpressed cells are depicted using IGV viewer. (**D**) Heatmap shown for CREB5 binding to SCL genes when LUC or CREB5 (C5) overexpressed LNCaP cells are cultured in Enzalutamide or DMSO.

To assess co-regulators of CREB5 in PC, we evaluated data from ChIP-sequencing and rapid immunoprecipitation and mass spectrometry of endogenous proteins (RIME) using the same LNCaP cell models [13, 14] that overexpressed *CREB5* or the negative control, *LUC*. In the RIME experiments, we also considered an engineered mutant form of CREB5, CREB5-L434P, which we characterized to have lost the ability to promote resistance to ARPI [13]. Compared to both negative controls, the RIME experiments indicated that CREB5 interacted with proteins that are AP-1 factors or known to be associated with them, including JUN, JUNB, JUND, ATF2, and ATF7 (Figure 4A). Based on motif enrichment analyses of the CREB5 binding site, we further observed significant enrichment of AP-1 binding motifs, including JUN, JUNB, and ATF2, which overlapped with our RIME results (Figure 4B). In the enzalutamide-treated context, CREB5-bound sites were still enriched near ATF2 motifs. Notably, while other enriched motifs were found, these were the only ones in which we noted consistent patterns of CREB5 distribution that were centered around these motifs, indicating that CREB5 binding patterns were highly consistent at ATF2 sites. When evaluating chromatin occupancy with and without enzalutamide, *CREB5*-overexpressing cells demonstrated CREB5 binding to the transcriptional start sites of several AP-1 factors, such as ATF3 and FOSL2 (Figure 4C). Furthermore, the transcriptional start site of FOSL1 had distinct CREB5-binding peaks as well, mostly in the enzalutamide-treated context. Finally, using the same ChIP-sequencing data, we evaluated CREB5 binding to SCL genes specifically. As highlighted by increased signal in the heatmaps, we found that CREB5 bound to the transcriptional start and end sites of all SCL genes defined by Tang et al. [7] to a greater degree than in negative control *LUC*-overexpressing cells (Figure 4D). These findings indicate that CREB5 regulates AP-1 transcription factors, of which many are SCL genes nominated by Tang et al.

### CREB5 overexpression drives tumor-forming phenotypes in prostate cancer cells

**Figure 5:**
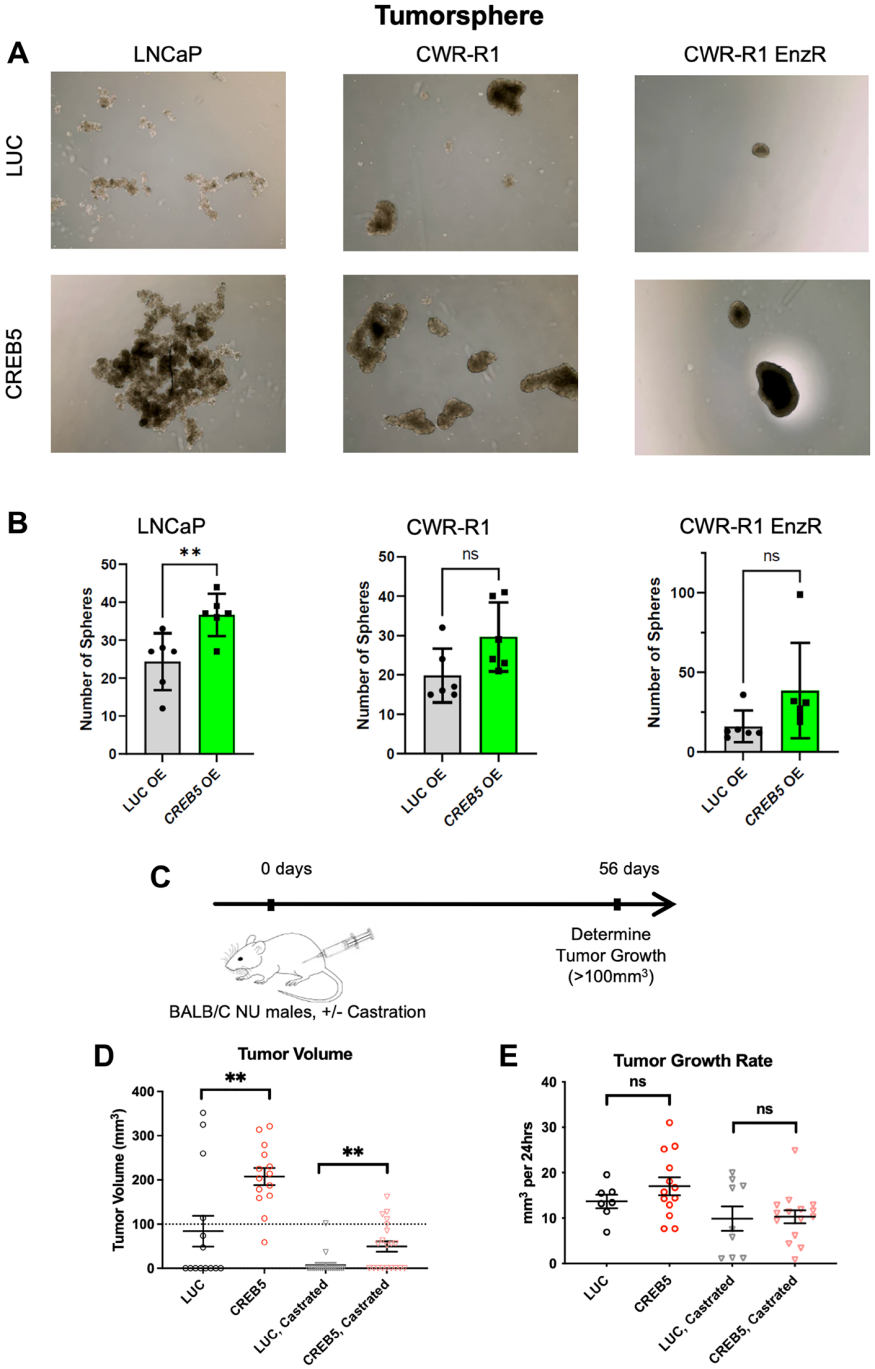
(**A**) Tumorsphere formation assay with six replicates performed in AR-positive prostate cancer (LNCaP), CRPC-like (CWR-R1), and CRPC-like enzalutamide-resistant (CWR-R1 EnzR) cell lines with CREB5 or LUC overexpression, (**B**) quantified by tumorsphere number with statistical comparisons done via Mann-Whitney tests. Error bars represent mean ± SD. ^*^p < 0.05, ^**^p < 0.01, ^**^p < 0.001, ^**^p < 0.0001, and ns = non-significant). (**C**) Schematic of the *in vivo* experimental timeline from 0 to 56 days in BALB/C NU male mice with or without castration. Endpoints were defined as tumor growth greater than 100 mm^3^. (**D**) Tumor volume and (**E**) tumor growth rate is depicted 56 days after CREB5 and LUC overexpressing LNCaP cell lines were implanted into castrated or non-castrated mice. Statistical comparisons were done using Mann-Whitney tests. ^*^p < 0.05, ^**^p < 0.001, and ns = non-significant.

Tumor cells with SCL traits are thought to have enhanced tumor-forming abilities. We thus preliminarily tested whether *CREB5* overexpression enhances the tumor-forming capacity of PC cells in a 3D tumorsphere assay using 3 distinct cell lines: LNCaP, CWR-R1, and CWR-R1 enzalutamide resistant (enzR). CWR-R1 is a castration-resistant PC line, whereas the CWR-R1 enzR is specifically resistant to enzalutamide as developed by Kregel et al. [26]. We found that *CREB5* overexpression promoted cell colony growth from a single cell in 3D culture, shown by a significant change in the total number of tumorspheres per well compared to *LUC* overexpressed cells (*p*-value < 0.01) in the LNCaP model (Figure 5A, 5B, Supplementary Figure 1 and Supplementary Data 1). However, we did not note this same pattern in the CWR-R1 or CWR-R1 enzR cell lines; we surmise the biological finding is due to CREB5 having the greatest impact on tumor cells that are hormone sensitive, as reported in our two prior studies [13, 14]. Furthermore, to better solidify CREB5’s possible tumorigenic properties, we examined the *in vivo* tumor volume of LNCaP cells with *CREB5* and *LUC* overexpressed. 56 days after implantation of LNCaP cells (Figure 5C), we noted that CREB5 overexpression significantly increased tumor volume in both castrated and non-castrated mice (*p*-value = 0.002 and 0.008, respectively; Figure 5D). Furthermore, there was no significant effect on the tumor growth rate (Figure 5E), which supports the hypothesis of CREB5 regulating SCL transcriptional programs and tumor promotion, rather than cell proliferation. Altogether, our results establish CREB5 as a gene that promotes tumorforming capacity in PC cells.

## DISCUSSION

In this study, we examined the role of *CREB5* in driving SCL transcription factors and tumor-promoting phenotypes in prostate cancer *in vitro* and *in vivo* models. While *CREB5* expression was inversely correlated with *AR* activity in CRPC, we importantly found that this reciprocal relationship also existed in primary PC. This relationship may implicate CREB5 in driving disease progression through transcriptional activity that resembles that of *KLF5* instead of *AR*. Furthermore, we noted gene expression analysis and modeling of gene behaviors revealed that *CREB5* had a tight positive association with *FOSL1*, a key AP-1 transcription factor previously implicated in stem cell features, therapy resistance, and metastasis [8, 9, 27]. In addition*, CREB5* was also associated with other SCL transcription factors. Through RIME and ChIP-sequencing, we found that CREB5 robustly bound and interacted with several AP-1 transcription factors, such as JUNB and ATF2. CREB5 also bound regulatory regions of several AP-1 genes, including ATF3 and FOSL2. Phenotypically, *CREB5* overexpression enhanced tumor-forming capacities of PC cell lines in 3D cultures and in mice, supporting the notion that *CREB5* expression also promotes aggressive traits. These findings nominate *CREB5* as a central regulator of lineage plasticity in PCs. While lineage plasticity is heavily implicated in advanced disease states and therapy resistance, our work suggests a mechanistic role of CREB5 in earlier stages of disease as well.

The inverse relationship between *CREB5* and *AR* activity across both primary PC and CRPC suggests that *CREB5* may engage in cellular activity that is agnostic of AR signaling. Clinically, while AR-positive PC remains the dominant subclass of CRPC that is resistant to ARPIs, collective work by Tang and Pitzen et al. highlights the importance of the SCL subtype of CRPC, which may be regulated by transcriptional activity of KLF5 [4, 7]. In CRPC and primary PC, we found robust positive correlations between CREB5 and KLF5 activity, as well as the transcription factors that regulate the SCL-CRPC transcriptional subtype [7], in both CRPC and primary disease. This finding aligns with recent discoveries of SCL features being present in primary tumors prior to cancer evolution due to therapeutic pressure [28]. The mechanistic relationship between CREB5 and KLF5 remains unclear, as we did not find direct evidence of their biochemical interactions through our RIME or ChIP-seq experiments. Regardless, it is possible that CREB5 predicts a mechanism of therapeutic escape that may manifest prior to PC treatment. We therefore hypothesize that high CREB5 activity in treatment-naïve contexts may act as a future biomarker for identifying patients at risk of ARPI resistance or progression to metastatic disease, and that the mechanism may be through underlying SCL traits.

Prior work from our group and others also implicates KLF5 as an SCL transcription factor that has been shown to be anti-correlated with *AR* signaling while supporting pro-metastatic transcriptional programs [29, 30]. Additionally, some studies suggest KLF5 drives a subset of CRPC tumors that do not express AR or canonical neuroendocrine features, deemed double-negative prostate cancers [30]. KLF5 has also been implicated in PC bone metastases specifically, where TGF-β signaling induces KLF5 acetylation, leading to bone metastatic lesions and chemoresistance [31]. Interestingly, KLF5 is not the only pro-metastatic transcription factor that CREB5 is strongly associated with in the clinical specimens. Our data indicate that *CREB5* is closely associated with *FOSL1* activity across both benign prostate tissue and CRPC specimens. The association between *CREB5* and *FOSL1* has potential implications beyond PC. While we investigated *FOSL1* as an SCL gene in the context of CRPC, *FOSL1*, an AP-1 transcription factor, has also been previously implicated in the metastasis of pancreatic cancers, as well as therapy resistance and poor patient outcomes in renal cell carcinomas [8, 9], highlighting its role as a regulator of aggressive tumor phenotypes. From this, our study suggests a broader regulatory relationship between *CREB5* and *FOSL1* expression, suggesting a potential regulatory axis that could contribute to lineage plasticity in PC as well as other malignancies.

Functionally, we found that *CREB5* overexpression perturbed the activity of multiple AP-1 genes in PC cells and drove tumor-forming phenotypes in 3D cell culture and larger tumor formation in mice. Given that AP-1 transcription factors regulate cell fate decisions across multiple cancers [32, 33], the interplay between CREB5 and these factors may not be restricted to PC and could extend to other malignancies with a propensity toward lineage plasticity and stemness-related traits. This appears to be the case in gliomas, as studies indicated that CREB5 was a top SCL gene based on several functional assays *in vitro* and *in vivo*, and also bound the AP-1 site of the key SCL gene *OLIG2* [18]. In addition, CREB5 is also associated with the progression of colorectal cancers and chemotherapy resistance [17, 34], in which AP-1 factors also serve as regulators of SCL traits [34–36]. While CREB5 has not been largely studied in melanomas, AP-1 factors are also known regulators of cancer cell plasticity [37]. Therefore, examining the mechanistic role of CREB5 in these cancers and how it regulates AP-1 factors may elucidate key mechanisms of cancer progression. Lastly, given that there are now anti-cancer therapies that target AP-1 factors [38], it may be worth considering if such agents perturb CREB5’s tumor-promoting activity and its interaction with AP-1 factors.

Taken together, this study indicates that CREB5 enhances PC tumor progression through genes that are associated with SCL traits. Therefore, CREB5 may act as a pivotal regulator of differentiation in both CRPC and primary PC. In cell lines, *CREB5* was associated with AP-1 family members, such as *FOSL1*, which may be a mechanism of such tumor progression. Altogether, increased CREB5 may lead to specific mechanisms of therapy response, and future therapeutic strategies may consider antagonizing CREB5 interactions with AP-1 complexes to mitigate deadly SCL phenotypes across cancer types.

## MATERIALS AND METHODS

### Expression analysis

SU2C/PCF 2019 [19] (*n* = 208) Fragments per Kilobase per Million mapped fragments (FPKM) data were downloaded from cBioPortal (https://www.cbioportal.org/) [39] and then converted to Transcripts per million (TPM). Genes with zero expression across all samples were removed from the data and duplicate gene names were made unique. The SU2C TPM data were further natural log-transformed for downstream analyses. TCGA-PRAD (*n* = 493) raw read counts and associated gene length information were downloaded using the TCGAbiolinks (version 2.30.4, project = “TCGA-PRAD”) R package. We then subsetted the raw reads to include only protein-coding genes and converted them to TPM. Genes with zero expression across all samples were removed from the data, and duplicate gene names were made unique. The TPM data were further natural log-transformed for downstream analyses. The GTEx Prostate dataset (*n* = 245) was downloaded directly from GTExPortal (https://www.gtexportal.org/home/) in units of TPM, so no further conversion was necessary. Genes with zero expression across all samples were removed from the data, and duplicate gene names were made unique.

### Alteration analysis

Samples were stratified into CREB5 High and Low groups based on the top and bottom 25% quartiles of *CREB5* expression. AR-V7 Capture Spliced Reads Per Million (SRPM) data were downloaded for the SU2C dataset from cBioPortal. Mann-Whitney U-tests were used to determine significance between the two groups in terms of gene expression and AR-V7 SRPM. Mutation and Copy Number Alteration data from TCGA-PRAD and SU2C were downloaded from cBioPortal. CNA data were labeled as HOMODEL, HETERODEL, NEUTRAL, GAIN, or AMP based on cBioPortal’s discrete CNA definitions. Samples that were found to have both a mutation and CNA other than NEUTRAL were labeled as “Multiple Alterations” for a given gene. Samples that were not present in the downloaded data were considered NA and removed from this analysis. Fisher’s exact test was used to determine significance between the CREB5 High and Low groups based on differences in alteration frequencies. Corrections for multiple comparisons were done where appropriate using the Benjamini-Hochberg method to control the FDR at a significance level of α equal to 0.05. For adjusted *p*-values: ^*^ if *q* ≤ 0.05 and *q* > 0.01, ^**^ if *q* < 0.01 and *q* ≥ 0.001, ^***^ if *q* ≤ 0.001 and *q* ≥ 0.0001, ^***^ if *q* ≤ 0.0001. ns if *q* > 0.05.

### Algorithm for linking activity networks

We utilized the Algorithm for Linking Activity Networks (ALAN) to depict the contextual relationships between genes of interest based on our prior study [25]. We utilized public datasets (SU2C and GTEx) for the inputs to our ALAN analyses and first converted all input RNA-seq data into TPM, as the transcript units were distinct. See public dataset preprocessing methods for additional details on each individual dataset. Visualizations were generated directly from distinct ALAN outputs for the GTEx, TCGA, and SU2C transcriptomic datasets. Heatmaps were used to depict the ALAN scores of two CRPC signatures (CRPC SCL and CRPC AR) [7] against *CREB5*, *FOSL1*, and *AR* in either SU2C or TCGA. ALAN networks of *FOSL1* and *CREB5* were extracted in two distinct contexts of prostate tissue, GTEx - benign prostate tissue and SU2C - mCRPC, and their comparison was depicted through cloud plots. Linear regression analysis was performed using GraphPad Prism software. R^2^ and *p*-values are reported.

### Generation of cell lines

LNCaP and CWR-R1 cell lines were purchased from the American Type Culture Collection (ATCC). CWR-R1 enzR were utilized from a prior study by Kregel et al. [26]. The human PC cell line LNCaP was cultured in RPMI medium 1640 (Gibco), supplemented with 10% FBS (R&D Systems), 0.2% GlutaMax (Gibco), and 1% Penicillin Streptomycin (Gibco). CWR-R1 cells were cultured in ATCC-modified 10% FBS RPMI-1640 (American Type Culture Collection). All cells were grown at 37° C and 5% CO^2^. To produce the *CREB5* and *LUC* overexpressing cells, during passage, 2 × 10^6^ cells in 4 mL single-cell suspension were mixed with 33 μL of either *CREB5* (VectorBuilder, ID: VB221028-1186bxp) or *LUC* (VectorBuilder, ID: VB240917-1425tbq) lentivirus and polybrene (Millipore Sigma; Cat# TR-1003) at a final concentration of 8 μg/mL. Cells were then plated in 25 cm^3^ tissue culture flasks (Fisher Scientific) and allowed to grow for 72 h. After attachment, cells then had media changed to ATCC-modified 10% FBS RPMI-1640 with 1 ug/mL puromycin (Gibco) and underwent selection for two passages alongside a mock transduction, which showed effective puromycin killing.

### RT-qPCR

Once the *CREB5* and *LUC* overexpressing CWR-R1 cells had been successfully established, all cells were plated in 10 cm^3^ dishes. After 48 h, RNA was harvested in 900 μL TRIzol reagent (Invitrogen; Cat# 15596026), needle homogenized and extracted using the RNeasy Plus Universal Mini Kit (Qiagen; Cat# 73404). Reverse transcriptase (RT) reactions were performed using 1 μg of purified RNA as a template using the High-Capacity cDNA Reverse Transcription Kit (Applied Biosystems; Cat# 4368814). The subsequent cDNA was diluted 5X in H^2^O and used for downstream applications. Expression levels of transcripts were quantified through qPCR with Fast SYBR^™^ Green Master Mix (Applied Biosystems; Cat# 4385612) on an QuantStudio 3 Real-Time PCR instrument (Applied Biosystems, Cat# A28131) according to manufacturer’s protocol using custom exon-spanning primers (250 nM) against *CREB5* (Integrated DNA Technologies, FW: 5′-ATTGACTCACCACCCTGCTG-3′, RV: 5′-GCATGAAGGTGGGAATGGGA-3′) and *FOSL1* (Integrated DNA Technologies, FW:5′-GGAGGAAGGAACTGA CCGACTT-3′, RV: 5′-CTCTAGGCGCTCCTTCTGCTTC-3′) and β-actin (Integrated DNA Technologies, FW: 5′-CACCATTGGCAATGAGCGGTTC-3′, RV: 5′-AGGTCTTTGCGGATGTCCACGT-3′). Change in threshold cycle (ΔCT) values was calculated for each sample compared to endogenous β-actin levels and compared to luciferase overexpressing control (ΔΔCT). All samples were performed in triplicate to determine mean standard error, and students’ *t*-tests were calculated with normalization to control to obtain *p*-values.

### Rapid immunoprecipitation mass spectrometry of endogenous proteins (RIME) and ChIP-sequencing

RIME interactions data were extracted from our previously published work [13], which is publicly available through supplemental tables in that study. These interaction data were then re-visualized via a heatmap generated using plotHEATmap. Similarly, ChIP-sequencing data were extracted from our previously published work [14] and can be accessed via GEO with the accession number: GSE137775. ChIP-sequencing data were also visualized using plotHEATmap to align all key binding sites, as well as using IGV viewer [40] to examine interactions at AP-1 binding sites for all ChIP-sequencing experiments.

### Tumorsphere experiments

*In vitro* floating spheroids were grown from 4 × 10^4^ cells (LNCaP, CWR-R1, and CWR-R1 EnzR) transduced with either lentiviral CREB5 (pLV(Exp)-EGFP:T2A:Puro-EF1A>hCREB5(NM_182898.4)/V5, Vector ID: VB221028-1186bxp, VectorBuilder) or luciferase control (pLV(Exp)-EGFP:T2A:Puro-EF1A>Luciferase/V5, Vector ID: VB240917-1425tbq, VectorBuilder) vectors. Transduced cells were selected using 1 μg/mL puromycin and cultured on ultra-low attachment 9.5 cm^2^ 6-well plates (Corning) and grown in suspension for 5 days. Tumorspheres were imaged using the EVOS^®^ XL Cell Imaging System and associated software (v1.4). Spheroids formed after 5 days were then transferred to standard-attachment 9.5 cm^2^ 6-well plates or 24 hours. Spheroids were then stained with crystal violet solution (Sigma-Aldrich), rinsed, and counted. Sphere formation was performed in sextuplicate.

### Mouse models and experimentation

We utilized mouse model data from a previous study of ours [14] that implanted cell xenografts subcutaneously into immunodeficient male BALB/C mice (RRID:IMSR_ TAC:balbnu). All procedures were performed under Dana-Farber Cancer Institute IACUC protocol 03-013. Here, tumor volume was calculated by using the ellipsoid formula, and results were re-visualized in PRISM. The Mann-Whitney test was used to calculate significance.

## SUPPLEMENTARY MATERIALS





## Abbreviations

ADTs: Androgen Deprivation Therapies
ALAN: Algorithm for Linking Activity Networks
AP-1: Activator Protein-1
AR: Androgen Receptor
ARPIs: Androgen Receptor Pathway Inhibitors
CRPC: Castration Resistant Prostate Cancer
PC: Prostate Cancer
SCL: Stem Cell-Like
