# Supplementary material for: CREB5 regulates stem cell-like transcriptional programs to enhance tumor progression in prostate cancer
Source: Oncotarget. 2026 Mar 17;17:59–73. doi: 10.18632/oncotarget.28850 (PMC13064935; doi:10.18632/oncotarget.28850)
Supplement: Supplementary file 1 [file oncotarget-26-049652-s001.pdf]

CREB5 regulates stem cell-like transcriptional programs to enhance tumor progression in prostate cancer

SUPPLEMENTARY MATERIALS

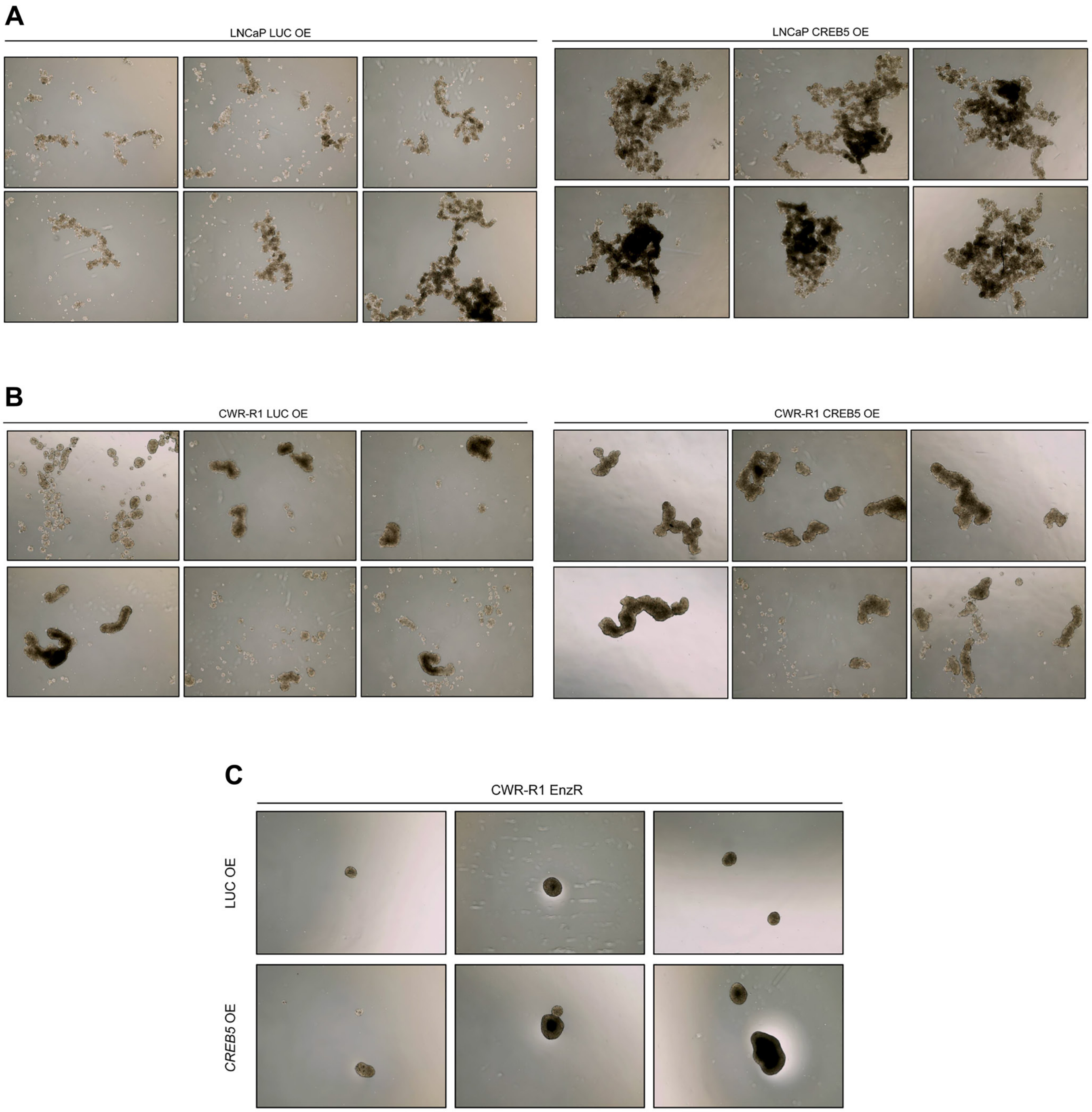

Supplementary Figure 1: Images depicting tumorsphere formation with either *CREBS* or *LUC* overexpression in (**A**) AR-positive (LNCaP), (**B**) castration-resistant (CWR-R1), and (**C**) castration-resistant enzalutamide-resistant (CWR-R1 Enz^R) cell lines.

Supplementary Data 1: See Supplementary Data 1
